# Predictive Factors for High Post-void Residual Volume in Older Females After OnabotulinumA Treatment for Severe Overactive Bladder Using a Machine Learning Model

**DOI:** 10.7759/cureus.42668

**Published:** 2023-07-29

**Authors:** Nobuo Okui, Tadashi Ikegami, Tatsuo Hashimoto, Yuko Kouno, Kaori Nakano, Machiko Aurora Okui

**Affiliations:** 1 Dentistry, Kanagawa Dental University, Kanagawa, JPN; 2 Diagnostic Imaging, Kanagawa Dental University, Kanagawa, JPN; 3 Internal Medicine, Kanagawa Dental University, Kanagawa, JPN; 4 Urology, Dr Okui's Urogynecology and Urology, Kanagawa, JPN; 5 Urogynecology, Yokosuka Urogynecology Clinic, Kanagawa, JPN

**Keywords:** urodynamic studies, support vector machine, random forest, post-void residual volume, machine learning models, frailty, overactive bladder, onabotulinuma, ai & robotics in healthcare

## Abstract

Introduction

Intravesical onabotulinumA injection is actively used for the treatment of overactive bladder (OAB). However, it occasionally results in significant post-void residual urine (PVR) volume, which can lead to complications and can further impair the activities of daily living in older people. Therefore, this study aimed to identify the predictors of a high post-onabotulinumA injection PVR volume in older women with severe OAB.

Methods

An observational study was conducted on older women who had previously received intravesical onabotulinumA injections to treat OAB between 2020 and 2022. Urodynamic studies and symptom assessments were conducted, and machine learning models, including random forest and support vector machine (SVM) models, were developed using the R code generated by Chat Generative Pre-trained Transformer 4 (ChatGPT, OpenAI, San Francisco, USA).

Results

Among 128 patients with OAB, 23 (18.0%) had a PVR volume of > 200 mL after receiving onabotulinumA injections. The factors associated with a PVR volume of > 200 mL were investigated using univariate and multivariate analyses. Age, frailty, OAB-wet, daytime frequency, and nocturia were significant predictors. Random forest analysis highlighted daytime frequency, frailty, and voiding efficiency as important factors. An SVM model incorporating daytime frequency, frailty, and voiding efficiency improved PVR volume prediction. Logit(p) estimation yielded an area under the receiver operating characteristic curve of 0.926294.

Conclusion

The study found daytime frequency, frailty, and voiding inefficiency to be significant factors associated with a PVR volume of > 200 mL, in older women with severe OAB. Utilizing advanced machine learning techniques and following the guidance of ChatGPT, this research emphasizes the relevance of considering multiple intersecting factors for predicting PVR volume. The findings contribute to our understanding of onabotulinumA injection treatment for OAB and support evidence-based decision-making using readily available information.

## Introduction

Overactive bladder (OAB) is a common urological condition in older individuals and is characterized by symptoms such as frequent urination, urgency, and urinary incontinence, caused by excessive bladder contractions [1]. OAB can significantly reduce an individual’s quality of life and has a significant impact on daily activities, particularly in individuals with frailty [2-4]. Furthermore, OAB medications often have strong side effects in older people, and the high incidence of physical disabilities and hospitalizations creates a vicious cycle [4].

In recent years, intravesical onabotulinumA injections have been actively used for the treatment of OAB [5]. This treatment inhibits excessive muscle contraction and improves symptoms [6]. However, it occasionally results in significant post-void residual urine (PVR) volume [6-8]. A large residual urine volume can lead to complications, such as urinary tract infections, and can further impair the activities of daily living in older people, potentially exacerbating frailty. Therefore, it is crucial to conduct research on the use of this treatment for OAB, that focuses on older patients with frailty.

In this study, we investigated factors that could predict a post-onabotulinumA injection PVR volume of > 200 mL in older women with severe OAB. Predictive modeling studies involving older individuals involve numerous complex factors [9]. Therefore, it is necessary to analyze data in a way that minimizes researcher bias. Consequently, we used Chat Generative Pre-trained Transformer 4 (ChatGPT; OpenAI, San Francisco, USA) to recommend the most efficient and accurate statistical methods for evaluating these predictive factors, using a machine learning model. ChatGPT excels in aiding research progression and analysis with its advanced natural language processing capability, supporting literature reviews, data analysis, and predictive model construction [10].

## Materials and methods

Study design and approval

This retrospective study included patients with OAB who received 100 U intravesical onabotulinumA injections (GlaxoSmithKline K. K., Tokyo, Japan) between 2020 and 2022. All patients provided written informed consent after receiving an explanation of the potential adverse events associated with these injections. The study was approved by the Regional Medical Ethics Committee (Ethical Review Board of Kanagawa Dental University 919/2023) and was registered in the UMIN Clinical Trials Registry (UMIN-CTR) under the registration number R000058728. The study database can be accessed at the Harvard Dataverse [11].

Patient selection criteria

Patients with OAB with the following criteria were included in this study: those who were treated with antimuscarinic and Beta3-adrenergic agonists therapy for a minimum of 4 months but showed resistance and continued to experience severe urgency incontinence, or those who had a minimum of one episode of urgent incontinence per day despite previous treatment with at least two antimuscarinic medications and one Beta3-adrenergic agonist. Patients with pelvic organ prolapse stage II or lower were included in the treatment group. Additionally, at the time of onabotulinumA injection, all patients were required to be free of urinary tract infections, intrinsic sphincter deficiency, or neurogenic bladder conditions.

Patients who were unable to provide informed consent, those who did not have a follow-up record for a minimum of 6 months after onabotulinumA injection, and those with severe mental illnesses that could potentially hinder the interview process were excluded. Three patients were excluded from the study due to reasons related to consent.

Urodynamic study

A urodynamic study was conducted using the Goby family of wireless urodynamic systems (EDAP TMS, Rhône, France) to evaluate detrusor overactivity, bladder outlet obstruction, and intrinsic sphincter deficiency. Regular urodynamic assessments were performed using the urethral pressure profile (UPP). Female participants with bladder outlet obstruction and decreased detrusor muscle activity were excluded. Bladder outlet obstruction was defined as evidence of a narrowed bladder outlet, a voiding detrusor pressure of > 35 cmH2O, a maximum flow rate (Qmax) of < 15 mL/s, or a voiding detrusor pressure of > 40 cmH2O. Patients were diagnosed with decreased detrusor muscle activity if the strength of contraction during voiding was ≤ 10 cmH2O and if they required abdominal straining or were unable to void. Various urodynamic parameters were meticulously measured and recorded, including the initial sensation of filling, bladder capacity during cystometry, bladder compliance, Qmax, PVR volume, detrusor pressure at Qmax (Pdet.Qmax), and bladder contractility index (BCI = Pdet.Qmax + 5 × Qmax). Additionally, voiding efficiency, calculated as the voided volume divided by the bladder capacity multiplied by 100%, was assessed.

Evaluation of OAB

During each clinic visit, multiple assessments were conducted, including the measurement of various parameters, such as voided volume, PVR volume, and Qmax. We calculated the daytime frequency within a 72-hour period (D72hFE) and the number of nocturia episodes based on a 3-day voiding diary. PVR volume measurements were performed using transabdominal ultrasonography. The bladder capacity was determined by combining the voided and PVR volumes. Urinary incontinence severity was evaluated using the International Consultation on Incontinence Questionnaire-Short Form [12], which considers the frequency, volume, and impact on daily life. Scores ranging from 1 to 5, 6 to 12, 13 to 18, and 19 to 20 represented mild, moderate, severe, and very severe incontinence, respectively. Additionally, the Overactive Bladder Symptom Score (OABSS) [13] was measured at each clinic visit to assess daytime and nighttime urinary frequency, urgency, and incontinence. The OABSS scores range from 0 to 15, with scores of < 5 indicating mild OAB, scores between 6 and 11 indicating moderate OAB, and scores of ≥ 12 indicating severe OAB. Furthermore, the presence of OAB-wet or OAB-dry was determined based on a 3-day voiding diary. Patients documented with at least one episode of urgent urinary incontinence were considered OAB-wet, whereas the remaining were considered OAB-dry. The Patient Perception of Bladder Condition score (PPBC) [14] is a self-assessment tool used to evaluate patients’ perception of their bladder condition and its impact on daily life, particularly for assessing symptoms related to an OAB. Patients rated their bladder condition on a scale of 0 (no problems at all) to 6 (many severe problems) to assess their own perception of their bladder condition. The degree of urgency was evaluated using a modified version of the validated Indevus Urgency Severity Scale (USS) [15]. This scale classifies the severity of urgency into four levels: 0, 1, 2, or 3 representing none, mild, moderate, and severe urgency, respectively. 

The Frailty questionnaire

The patients were also interviewed using the Frailty questionnaire [16], which assessed Fatigue, Resistance, Ambulation, Illnesses, and Loss of weight. Each item was rated on a scale of 1-5, and scores of 1-2 or ≥ 3 out of 5 indicated pre-frailty and frailty (FRAILTY), respectively. 

Large post-void residual volumes

The duration of the sustained large PVR volume interval was measured from the day when the first large PVR volume was recorded during a follow-up visit until the day when it was documented that no large PVR volume was present, and subsequently until the last follow-up visit. Following the previous literature, a PVR volume of > 200 mL was considered large [17].

Statistical analyses

Continuous variables are reported as means ± standard deviations or as numbers and percentages. The data were used to provide statistical guidance and generate R language codes with the assistance of ChatGPT. Statistical analyses were performed using R statistical software version 2.15.1 (R Core Team, Austria, Vienna). Univariate regression analyses included variables such as age, sex, OAB-wet or OAB-dry classification, comorbidities, OABSS, USS score, 3-day voiding diary data, and urodynamic variables. Multivariate stepwise logistic regression analysis was conducted using variables selected by ChatGPT with a significance threshold of p < 0.25 from the univariate analysis. A significance level of p < 0.05 was considered statistically significant. Receiver operating characteristic (ROC) curve analysis was performed to identify the optimal cutoff value using the values derived from the logit (p) equation. ChatGPT generated an R code for machine learning, resulting in the development of random forest and support vector machine (SVM) models. Through this learning process, the significant factors and their corresponding conditions were identified. ROC curve analysis was performed to identify the optimal cutoff value for predicting a significant PVR volume of > 200 mL. A multivariate logistic regression analysis incorporating all significant factors was performed to predict the probability (p) of a significant PVR volume and to obtain logit transformation (logit(p)). The optimal cutoff value was determined based on the point on the ROC curve closest to the upper-left corner.

## Results

Patients

**Figure 1:**
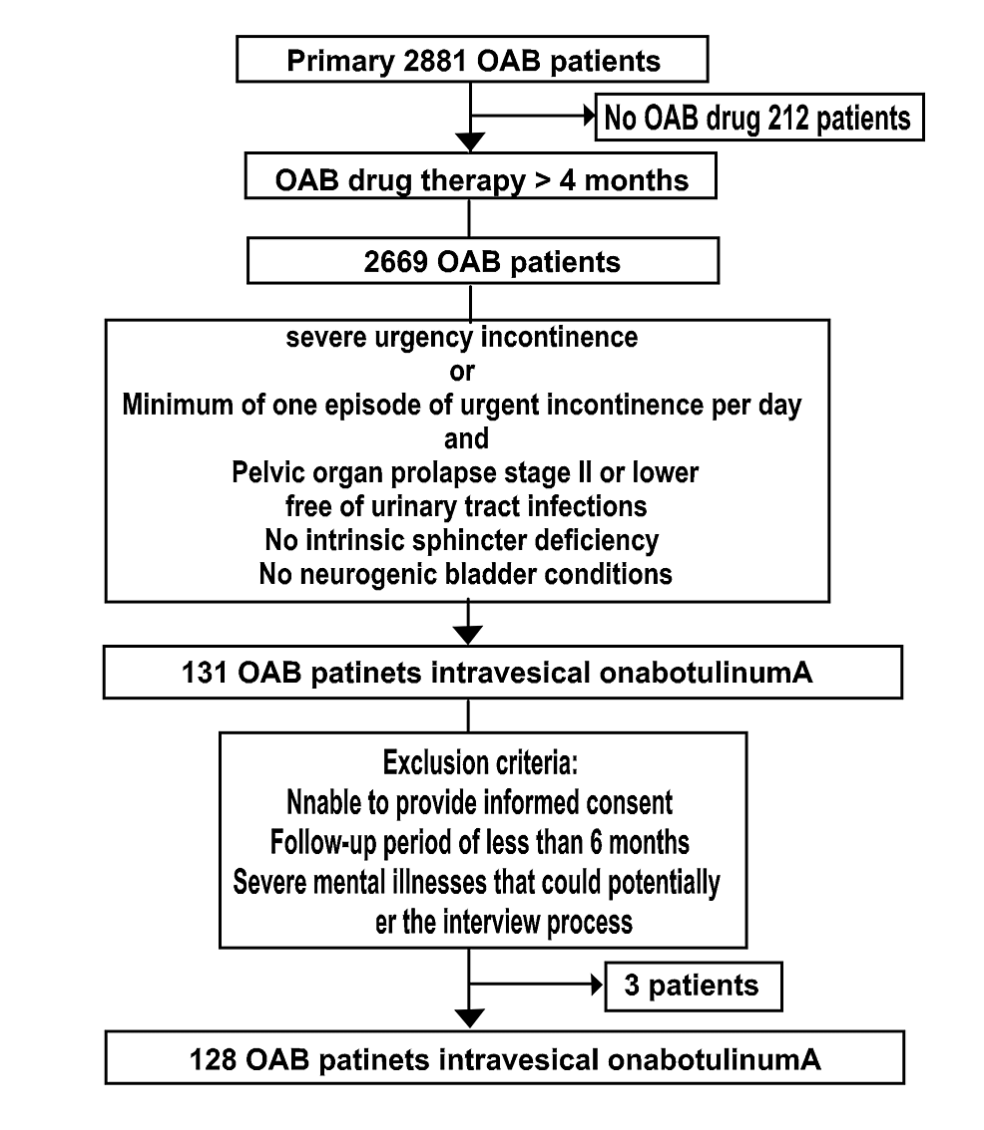
Figure 1. Flowchart showing the study criteria OAB: Overactive bladder

Figure 1 shows the study flowchart. During the study period, 2,881 women with OAB sought medical consultation. Among them, 212 individuals did not require OAB medication. Out of the patients who took OAB medication for more than 4 months, 131 were eligible for intravesical onabotulinumA injections. During the 6-month follow-up period, our study included 128 females (Age: 44-91 years, Median: 72.5 years). 

No dropouts occurred during the study period. In this cohort, 23 (18.0%) female patients experienced a PVR volume of > 200 mL. To investigate the factors influencing the likelihood of the PVR volume exceeding 200 mL, we used appropriate statistical methods, including logistic regression for univariate analysis and logistic multivariate analysis for multivariate analysis, with the assistance of ChatGPT.

Univariate correlation of various factors

In univariate analysis, we analyzed factors associated with a PVR volume of > 200 mL in patients with OAB. Logistic regression analysis, guided by ChatGPT, revealed several noteworthy findings. Age and FRAILTY showed positive correlations with increased odds of having a PVR volume of > 200 mL (age: odds ratio = 1.06, 95% CI: 0.978-1.050, p = 0.5270; FRAILTY: odds ratio = 2.72, 95% CI: 2.89-3.29, p = 0.0024). Conversely, the presence of OAB-wet exhibited a slight decrease in these odds (odds ratio = 0.234, 95% CI: 0.072-0.758, p = 0.0155). OAB-wet and OAB-dry were considered as reference values because of the presence of one individual with unstable memory. Additionally, each additional D72hFE and nocturia episodes demonstrated significant associations with increased odds of having a PVR volume of > 200 mL (D72hFE: odds ratio = 3.69, 95% CI: 2.69-9.85, p = 0.0536; nocturia: odds ratio = 1.79, 95% CI: 1.09-2.95, p = 0.0225). These findings underscored the importance of considering age, FRAILTY, OAB-wet status, D72hFE, and nocturia episodes when assessing the risk of having a PVR volume of > 200 mL.

Consideration of correlations before conducting multivariate analysis

Before conducting multivariate analysis, we performed preprocessing to address variables that potentially had correlations, such as age and FRAILTY. As ChatGPT did not provide specific suggestions in this regard, we made the selection based on human judgment. The correlation coefficients among age, FRAILTY, OAB-wet, D72hFE, nocturia episodes, voiding efficiency, cystometric bladder capacity, and BCI are as follows:

The correlation coefficient between age and FRAILTY was 0.2819 (moderate positive correlation). The correlation coefficients between age and each variable were as follows: age and OAB-wet (-0.1949), age and D72hFE (0.2908), age and nocturia (0.1497), age and voiding efficiency (-0.2086), age and cystometric bladder capacity (-0.2661), and age and BCI (-0.054). The correlation coefficient between voiding efficiency and BCI was 0.088 (weak positive correlation). 

If there are high correlation coefficients among the endpoints, it is recommended to exclude one of the variables with a high correlation for the multivariate analysis. In this case, we determined that there is a moderate positive correlation between age and FRAILTY, but the correlation between voiding efficiency and BCI is weak and not particularly high. Taking these findings into consideration, we concluded that conducting multivariate analysis excluding age would minimize the risk of multicollinearity.

Multivariate correlation of selected various factors

FRAILTY and D72hFE emerged as significant predictors in the multivariate model. Table 1 presents the results of a logistic multivariate analysis conducted using ChatGPT, which selected parameters based on univariate analysis. According to the multivariate analysis, the following variables exhibited significant associations with the likelihood of having a PVR volume of > 200 mL. Patients with FRAILTY had significantly higher odds of experiencing a PVR volume of > 200 mL than those without FRAILTY (odds ratio = 30.3, 95% CI: 3.44-267.0, p = 0.0020). Each additional D72hFE was significantly associated with an increased likelihood of having a PVR volume of > 200 mL (odds ratio = 1.97, 95% CI: 1.97 1.38-2.82, p < 0.001). An increase in the number of nocturia episodes within a 72-h period showed a tendency toward an elevated likelihood of having a PVR volume of > 200 mL, although this did not reach statistical significance (odds ratio = 2.65, 95% CI: 0.997-7.07, p = 0.051). The remaining variables listed in Table 1 did not exhibit significant associations with the likelihood of having a PVR volume of > 200 mL on multivariate analysis.

**Table 1:** Results of a logistic multivariate analysis conducted using ChatGPT to select parameters based on univariate analysis BCI: Bladder contractility index, CAD: Coronary artery disease, CHF: Congestive heart failure, CKD: Chronic kidney disease, COPD: Chronic obstructive pulmonary disease, FRAILTY: Pre-frailty and frailty, ICIQ-SF: International Consultation on Incontinence-Questionnaire–Short Form, OAB-dry: Overactive bladder without incontinence, OABSS: Overactive Bladder Symptom Score, OAB-wet: Overactive bladder with incontinence, Pdet.Qmax: detrusor pressure at Qmax, PPBC: Patient perception of bladder condition, D72hFE: daytime frequency within a 72-hour period, USS: Urgency severity scale, UUI: Urgency urinary incontinence

Variable	Values	Univariate		Multivariate	
		Odds ratio	p	Odds ratio	p
Age (years)	71.8 ± 12.9	1.06 (1.010–1.120)	0.024		
FRAILTY	26 (18.0%)	110.0 (25.4–477.0)	< 0.001	28.4 (3.37-239.0)	0.0021
OAB-wet #	114 (89.0%)	0.234 (0.072–0.758)	0.0155	2.15 (0.087– 52.7)	0.640
OAB-dry #	24 (11.0%)	—	—	—	—
CKD	8 (6.25%)	3.0 (0.663–13.6)	0.154		
COPD	6 (4.69%)	0.089 (-0.0861–0.258)	0.319		
CAD	45 (35.1%)	2.40 (0.413–14.0)	0.329		
Diabetes mellitus	40 (31.3%)	0.955 (0.359–2.540)	0.926		
CHF	26 (18.0%)	0.953 (0.291–3.120)	0.937		
Parkinsonism	1 (0.78%)	—	—	—	—
Dementia	6 (4.67%)	0.909 (0.101–8.170)	0.932		
USS	2.91 ± 0.62	0.997 (0.484–2.05)	0.993		
PPBC	4.84 ± 0.60	0.967 (0.454–2.06)	0.930		
D72hFE	20.5 ± 4.88	3.69 (1.63–8.36)	0.0012	1.69 (1.05-2.70)	0.029
Nocturia episodes (72 h)	5.81 ± 2.75	1.25 (1.040–1.52)	0.020	2.67 (0.963-7.38)	0.05
UUI episodes (72 h)	9.82 ± 1.25	0.998 (0.69–1.43)	0.993		
Maximum flow rate (mL/s)	10.24 ± 1.19	1.010 (0.691–1.47)	0.962		
Voided volume (mL)	214 ± 35.5	0.988 (0.974–1.000)	0.077		
Post-void residual (mL)	40.1 ± 14.9	1.030 (0.994–1.060)	0.117		
Bladder capacity (mL)	255 ± 36.2	0.999 (0.987–1.01)	0.917		
Voiding efficiency (%)	83.7 ± 6.62	0.893 (0.893–0.962)	0.002	0.989 (0.84-1.16)	0.89
First sensation of filling (mL)	116 ± 11.7	1.000 (0.962–1.04)	0.997		
Cystometric bladder capacity (mL)	222 ± 31.1	0.98 (0.961–0.999)	0.040	0.989 (0.94-1.03)	0.59
Detrusor overactivity	128 (96%)	0.647 (0.0643–6.52)	0.712		
Pdet.Qmax (cmH_2_O)	27.4 ± 8.52	1.00 (0.950–1.060)	0.956		
BCI	94.4 ± 22.4	0.954 (0.934–0.975)	< 0.001	1.01 (0.96-1.05)	0.71
OABSS	12.3 ± 1.59	1.010 (0.758–1.34)	0.968		
ICIQ-SF	14.8 ± 2.73	1.110 (0.943–1.31)	0.208		

Variable selection using ChatGPT for comprehensive analysis

Based on the results of multivariate and univariate analyses, ChatGPT proposed the selection of variables for further analysis. Multivariate analysis identified the following variables: FRAILTY, D72hFE, and nocturia episodes within 72 h as predictors. Additionally, univariate analysis suggested the inclusion of age, BCI, voiding efficiency, and cystometric bladder capacity. Therefore, we conducted a comprehensive analysis using these variables.

Feature importance in random forest model

ChatGPT recommended methods, such as random forest and SVM models for machine learning, to evaluate specified features. We thus conducted a random forest analysis on the complete dataset, consisting of 128 samples and seven features, to determine the factors with the highest predictive importance. The feature importance results presented in Table 2 are calculated based on the mean squared error increase (%IncMSE) and node purity increase (IncNodePurity) measures. When the target variable was considered as a categorical value, the importance results are shown based on the IncNodePurity. However, when the target variable was considered as a continuous value, the importance results are shown based on the %IncMSE.

**Table 2:** Random forest variable importance BCI: Bladder contractility index, FRAILTY: pre-frailty and frailty, D72hFE: daytime frequency within a 72-hour period

Predictor	%IncMSE	IncNodePurity
Age	0.0044769	1.8020206
FRAILTY	0.0360636	2.8095423
D72hFE	0.0858477	6.3093305
Nocturia episodes (72 h)	0.0111649	1.3528381
BCI	-0.000607	0.6661288
Voiding efficiency	0.0090423	2.3126757
Cystometric bladder capacity	0.0035157	1.5981565

As shown in Table 2, based on the feature importance results, we identified the top-three factors that exhibited the highest predictive importance. The most influential factor was D72hFE with a %IncMSE value of 0.0858 and an IncNodePurity value of 6.3093. Next, FRAILTY demonstrated significant importance, with a %IncMSE of 0.0361 and an IncNodePurity of 2.8095. The third most important factor was age, with a %IncMSE value of 0.0045 and an IncNodePurity value of 1.8020. These three factors (D72hFE, FRAILTY, and voiding efficiency) emerged as key predictors that significantly contributed to the predictive performance of our model.

Feature transformation and improved SVM performance

We analyzed a dataset that included three explanatory variables: D72hFE, FRAILTY, and voiding efficiency, using an SVM model to predict the occurrence of a PVR volume of > 200 mL. The SVM model was configured with the following parameters: SVM-Type as eps-regression, SVM-Kernel as radial, cost as 1, gamma as 0.3333333, and epsilon as 0.1. Forty-six support vectors were identified in the dataset to evaluate the predictive performance of the model. These results suggested that the three explanatory variables play important roles in predicting the PVR volume. The predictive performance for the PVR volume was improved by optimizing the parameters of the SVM model and selecting support vectors.

Estimating logit(p) and optimal cut-off values

The estimated logit(p) for a given baseline D72hFE (a), FRAILTY (b), and voiding efficiency (c) can be represented as

logit(p) = (-15.28481) + (0.6358258 * a) + (1.178545 * b) - (0.06345937 * c)

**Figure 2:**
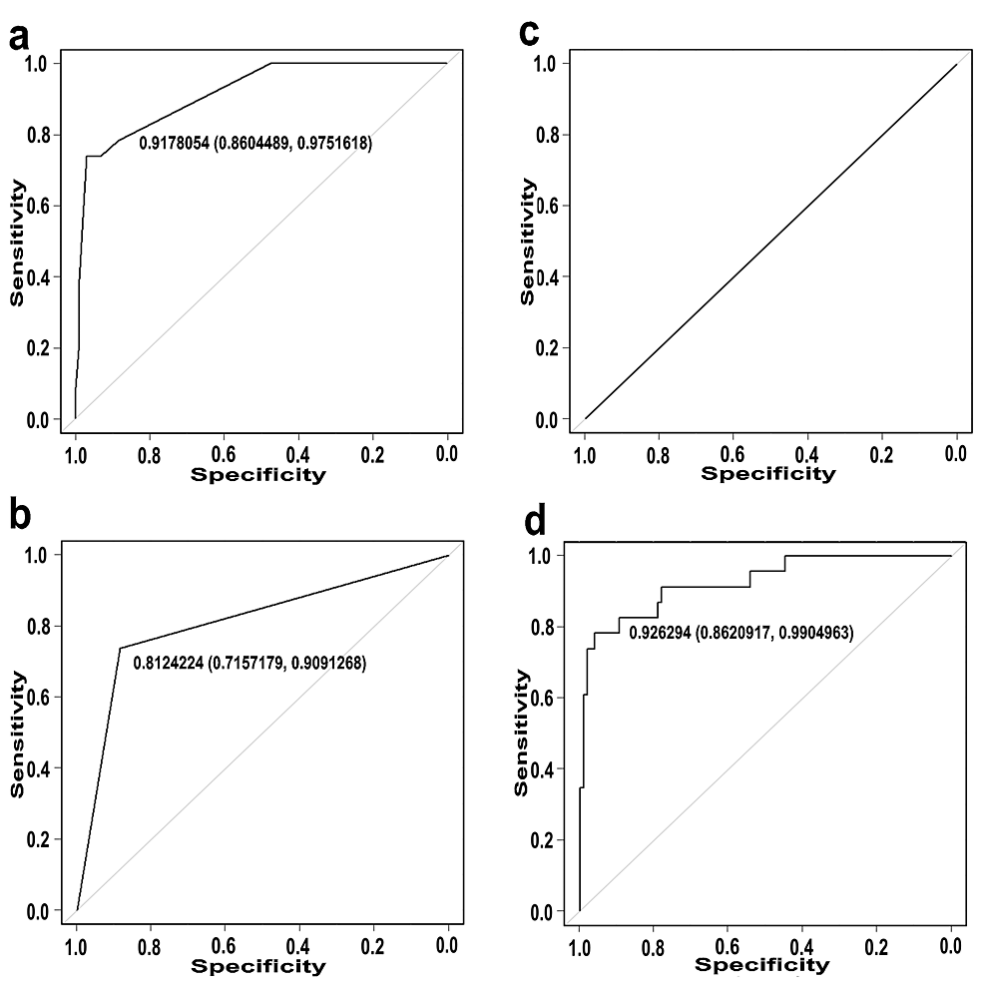
ROC curves and performance metrics The vertical axis represents the sensitivity ranging from 0 to 1.0, and the horizontal axis represents the specificity ranging from 1.0 to 0.0. Each graph (a: D72hFE, b: FRAILTY, c: voiding efficiency, d: logit(p)) shows the ROC curves for the association between the respective variables and PVR. The AUC (95% confidence interval) is indicated in each graph. D72hFE: daytime frequency within a 72-hour period, FRAILTY: pre-frailty and frailty, PVR: post-void residual urine, ROC: Receiver operating characteristic.

Figure 2 shows the ROC curves of the baseline D72hFE (Figure 2a), FRAILTY (Figure 2b), voiding efficiency (Figure 2c), and logit(p) (Figure 2d). For D72hFE, the area under the ROC curve (AUC) was 0.9178054 (95% CI = 0.8604489-0.9751618). The sensitivity ranged from 0.04% to 100% and the specificity ranged from 0% to 100% for D72hFE (Figure 2a). For FRAILTY, the AUC was 0.8124224 (95% CI = 0.7157179-0.9091268). The sensitivities were 100%, 73.91%, and 0%, respectively, and the specificities were 100%, 11.43%, and 0%, respectively (Figure 2b). For voiding efficiency, the AUC was 0.5 (95% CI = NA to NA). The sensitivities were 100% and 0%, respectively, and the specificities were 100% and 0%, respectively (Figure 2c). For logit(p), the AUC was 0.926294 (95% CI = 0.8620917-0.9904963). The sensitivity and specificity were 91.3% and 92.3%, respectively (Figure 2d).

## Discussion

Similar to our study, previous studies [17,18] have investigated and identified the important predictors of adverse events, such as increased PVR volume following intravesical onabotulinumA injections. Hsiao et al. [17] used logistic univariate and multivariate analyses, similar to our approach, and identified daytime frequency episodes and voiding efficiency as significant predictors using logit(p) and ROC analyses. However, the selection of these variables by Hsiao et al. was based on the logistic regression analysis results of Jiang et al. [18]. In addition, Abrar et al. reported that, in female patients, uterine removal was identified as a risk factor through multivariate logistic regression analysis** **[19]**.** One notable distinction between these previous studies and ours was the inclusion of frailty as a predictor.

Before administering onabotulinumA injections to all patients, we planned our treatment approach and collected patient data based on the studies by Hsiao et al. [17] and Jiang et al. [20]. One important distinction between previous studies and our research was the higher mean age of the study population in our study, which was 71.8 ± 12.9 years, specifically focusing on older women. In previous studies comparing a mixed group of elderly individuals (82.0±4.6 years) with a younger group (58.8±11.9 years) [21], PdetQmax and diabetes mellitusmedical history were associated with urinary retention.

Previous female patients' reports on adverse events related to the PVR volume following onabotulinumA injections have reported mean ages of 65.5 years [22], 37.5 ± 15 years [23], 68 years [17], and 64 ± 13.2 years [18]. This highlights the uniqueness of our study, which focused exclusively on the older female population. Considering the natural decline in overall muscle strength that occurs with age, we considered an investigation of FRAILTY to be essential. Therefore, we added factors, including FRAILTY and components of the Frailty questionnaire. The relationship between frailty and OAB has been extensively studied [22, 23], with some reports suggesting that frailty worsens OAB symptoms [24]. Furthermore, some findings have suggested that treatments targeting frailty may have beneficial effects on OAB [25].

Rather than relying on previous studies to select important factors, we aimed to eliminate human bias and explore each factor's significance from a different perspective. ChatGPT assisted in factor selection and subsequent analyses. Univariate and multivariate logistic analyses yielded similar conclusions to that in the study by Hsiao et al. [17], with the notable addition of frailty as a factor.

The random forest and SVM methods proposed by ChatGPT have advantages in evaluating explanatory variables for the target variable. Random forest models handle complex interrelationships among factors and evaluate feature importance, making them suitable for high-dimensional or feature-rich datasets like intravesical onabotulinumA injections. SVM models handle different scales of explanatory variables and allow for selecting appropriate hyperparameters, making them optimal for our study.

Random forest and SVM methods have also been used in OAB research. Sheyn et al. developed a random forest model to predict the response to anticholinergic drugs in patients with OAB [26], whereas Zhou et al. evaluated the urinary microbiota in female patients with OAB using an SVM model [27]. 

According to the ROC analysis, the AUC for D72hFE in our study was 0.9178054 (95% CI = 0.8604489-0.9751618). The sensitivity ranged from 0.04% to 100%, and the specificity ranged from 0% to 100%. In Hsiao et al.'s study, the ROC curve had an area of 0.72 (95% CI = 0.60-0.84), with a sensitivity of 73.0% and specificity of 65.6% [17]. Jiang et al. [20] identified D72hFE as an important predictor. On the basis of these findings, it is evident that this factor is an important predictor of the PVR volume. However, the importance of voiding efficiency may not be as pronounced as that of D72hFE. D72hFE, FRAILTY, and voiding efficiency are easily assessed in an outpatient setting. Therefore, investigating these three factors could provide valuable information for the management of OAB in older patients.

Our study may have limitations in terms of data quality, potential biases, and generalizability. Although advanced machine learning techniques and ChatGPT have been used here, the accuracy and interpretation of the results should be considered. It is important to acknowledge that older women with high PVR volumes may be influenced by detrusor insufficiency, which could significantly impact the observed results, apart from the effects of onabotulinumA injections. While our study focused on the predictors of high PVR volume following onabotulinumA injections, detrusor insufficiency should also be taken into account as a potential confounding factor that might influence the outcomes. The interplay between detrusor insufficiency and the effects of onabotulinumA injections warrants further investigation to better understand the individual contributions of each factor to the observed results.

## Conclusions

In conclusion, our study investigated the predictive factors associated with a post-intravesical onabotulinumA injection PVR volume of ≥ 200 mL in older women with severe OAB. The analysis, guided by ChatGPT, revealed several significant predictors, including age, FRAILTY, wet-type OAB, daytime frequency episodes, and nocturia episodes, of having a PVR volume of > 200 mL. D72hFE and FRAILTY emerged as independent predictors in the multivariate analysis, whereas D72hFE and FRAILTY were identified as significant factors in the random forest model. The SVM model demonstrated an improved performance through feature transformation. These findings provide valuable insights into predicting a PVR volume of > 200 mL in older patients with severe OAB and highlight the importance of considering multiple factors and employing advanced machine learning techniques in research and clinical decision-making. Larger prospective studies with standardized data collection methods are necessary to validate and generalize these findings.
